# Squamous cell carcinoma of the breast: a case report

**DOI:** 10.4076/1757-1626-2-7336

**Published:** 2009-07-10

**Authors:** Roberto Murialdo, Davide Boy, Yuri Musizzano, Lucia Tixi, Federica Murelli, Alberto Ballestrero

**Affiliations:** 1 Department of Internal Medicine, S. Martino Hospital, University of Genoa, Viale Benedetto XV n.6 Genova, Italy; 2 Department of Surgical and Morphological Disciplines and Integrated Methodologies, Division of Pathology, University of Genoa, Viale Benedetto XV n.6 Genova, Italy; 3 Department of Surgery, Breast Unit, S. Martino Hospital, University of Genoa, 16132, Italy

## Abstract

**Introduction:**

Pure primary squamous cell carcinoma of the breast is uncommon and it's debated the correct management of this disease.

**Case presentation:**

A 54-years-old woman presented with signs and symptoms of mastitis of left breast. A palpable well circumscribed and firm mass, measuring about 40 mm, was present in the left lower lateral quadrant. She underwent antibiotic therapy without benefit. She performed an ultrasound and mammographic scan of the left breast. Fine needle aspiration cytology revealed an infiltrative poorly differentiated squamous cell carcinoma. Total body CT scan and bone scan excluded distant metastasis. Subsequently wide local excision of the left breast with ipsilateral axillary lymph nodes dissection was performed. The pathological examination revealed an infiltrative poorly differentiated squamous cell carcinoma of the breast. Adjuvant chemotherapy cisplatin and 5-fluorouracil based was administered. Patient refused locoregional radiotherapy. Twenty-eight months after surgery the patient was disease free.

**Conclusions:**

Pure primary squamous cell carcinoma of the breast is a rare and aggressive disease often treatment-refractory. An optimal systemic treatment is needed to improve patient's outcome.

## Introduction

Pure primary squamous cell carcinoma of the breast is a relatively uncommon desease. It is diagnosed if more than 90 % of the malignant cells are squamous. It represents less than 0.1 % of all breast carcinomas [1].

The prognosis of this type of breast cancer is still a subject of controversy; some reports suggest that it is aggressive, with an outcome comparable to poorly differentiated ductal carcinoma of the breast [2]-[5].

Clinical and radiologic appearances are not specific to discriminate malignant versus benign lesions or to characterize the hystotype.

An extensive review of the literature reveals about fifty reports and two important series of pure squamous cell carcinoma of breast strictly following the Macia et al. diagnostic criteria [6]: thirty-three patients by M.D. Anderson Cancer Center and eleven patients reported by a Spanish group [7,8].

Macia and colleagues defined pure squamous cell carcinoma as a tumor with following characteristics [6]:

1.Â No other neoplastic components, such as ductal or mesenchymal elements, are present in the tumor.

2.Â The tumor origin must be independent from the overlying skin and nipple.

3.Â Absence of an associated primary squamous cell carcinoma in a second site.

In this report, we present a patient who initially appeared with signs and symptoms of mastitis and underwent to antibiotic therapy without benefit.

Histologic examination of the tissue obtained from surgery revealed a pure primary squamous cell carcinoma of the breast.

## Case presentation

The patient was a 54-year-old Italian Caucasian female. She was a non-smoker. She was evaluated in our hospital for recent onset of pain and tenderness in the left breast. She referred swelling of the breast, but denied nipple discharge. Her past medical history was not significant. Her family history was not significant for breast cancer; she denied oral contraceptives use in the past and she give birth to one child. Her most recent Pap smear had been negative for any squamous lesion. Physical examination revealed erythema, hyperaemia and tenderness to touch of the left breast. A palpable well circumscribed and firm mass measuring about 40 mm was present in the left inferior lateral quadrant. The right breast examination was normal. There was no evidence of lymphadenopathy on physical examination, including the axillary and supraclavicular regions.

She underwent an ultrasound examination of the left breast, which revealed a defined 29 mm mass with a thick rind and reduced central echogenicity, consistent with a cystic space. Mammography showed a round, high-density mass with almost regular but partially irregular margins, measuring approximately 30 mm.

Fine needle aspiration cytology revealed an infiltrative poorly differentiated squamous cell carcinoma.

Total body CT scan, bone scan, laboratory data (blood count, serum electrolytes, liver function tests, creatinine, prothrombin and partial thromboplastin time), tumors markers, including the levels of serum carcinoembryonic antigen, carbohydrate 15-3 and squamous cell carcinoma antigen were within normal range.

**Figure 1:**
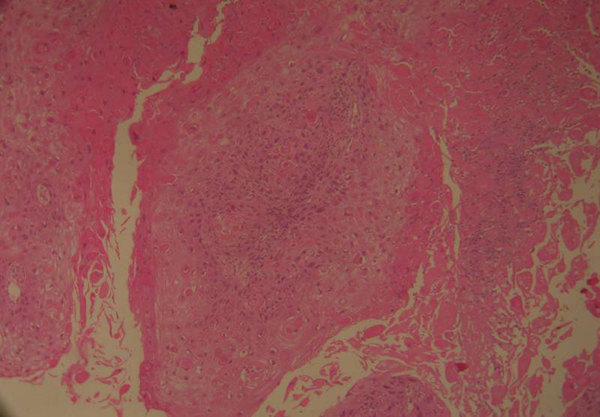
**Large Squamous cells with keratinising eosinophilic glassy**. It's present a modest inflammatory intralesional reaction (Hematoxylin-Eosin, 20x).

**Figure 2:**
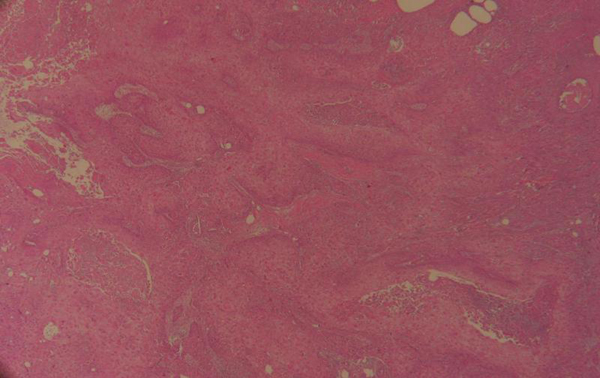
**Solid structure with central cystic space containing keratin and necrotic debris (Hematoxylin-Eosin, 10x)**.

The patient underwent a wide local excision of the left breast with ipsilateral axillary lymph nodes dissection. Gross examination revealed a 4.7 cm tumor with central cystic space located in the center to lower outer quadrant of the left breast. There were no component of obvious invasive ductal carcinoma or other features of metaplastic carcinoma for example spindle cells, or osseous or cartilaginous metaplasia. It revealed large squamous cells with keratinizing eosinophilic glassy (Figure 1). The lesion tends to be differentiated sufficiently to form keratohyaline granules. The central cystic space contain keratin and necrotic debris (Figure 2). Vascular or neural invasion was not identified. There was no involvement of the skin or skin adenexae of the breast or the nipple. The uninvolved mammary parenchyma contained fibrocystic changes. Seventeen axillary lymph nodes were dissected and they were negative for metastatic disease. Immunohistochemical evaluation was negative for estrogen receptor (ER), progesterone receptor (PgR) and Her2/neu overexpression and positive for EGFR. Ki-67 proliferation index was unfavourably high at 60%.

The patient received an adjuvant therapy based on 5-Fluouracil 600 mg/mq day 1 and Cisplatin (CDDP) 25 mg/mq days 1, 2, 3 every 3 weeks for six cycles. Afterwards patient refuse radiation therapy to the residual left breast.

Twenty-eight months after surgery the patient was asymptomatic and disease free.

## Discussion

Pure primary squamous cell carcinoma of the breast is a rare condition and is considered to arise through metaplastic change of ductal carcinoma cells [9].

The concept of a disease continuum with varying degrees of squamous metaplasia was supported by Stevenson et al. who conclude that SqCC mostly represents an extreme form of squamous metaplasia within adenocarcinoma [9]. An alternate theory is that arise directly from the epithelium of the mammary ducts.

The SqCC of the breast are generally large (> 4 cm) at diagnosis and cystic in 50% of the cases [10].

The prognosis of this type of breast cancer is still regarded as somewhat controversial, though many studies suggest that it is an aggressive disease that may behave like poorly differentiated breast carcinoma [1,5,11].

The breast SqCC is usually a high-grade and hormone receptor-negative tumor [3]. This means that hormone-based therapy may not be effective in these tumors.

HER2/neu is also usually not over-expressed or amplified in this disease [12]. The high frequency of EGFR positivity is interesting and may be exploited in the development of future treatments.

No findings on mammography are specific for this diagnosis and this may explain the advanced disease stage at diagnosis [13]. Breast ultrasound has been reported to be more helpful with these tumors appearing as solid hypoechogenic masses with complex cystic components [14].

The treatment of SqCC of the breast does not differ from other common histological types of brest cancer and may involve surgery, chemotherapy, hormonal therapy and radiation therapy. Because of its rarity the most appropriate therapeutic regimen for SqCC of the breast is still unclear.

A recent literature review reveals that an average of 70% of patients with SqCC of the breast do not present axillary lymph nodes involvement but due the unpredictable lymph nodes dissemination, axillary lymph nodes dissection could always be performed for staging purposes [3].

As a result of lack of data, the issue of whether to prescribe adjuvant treatment for SqCC of the breast, remain unsolved [3]. A little contribution derived from a review by M.D. Anderson group of clinical pathologic features, management and outcome of SqCC of the breast in a series of 33 patients. Nineteen of the 31 patients with localized disease received adjuvant chemotherapy and 5 patients received neoadjuvant chemotherapy. The neoadjuvant regimens used were antracycline/taxane-based for four patients and anthracycline-based for one patient. The tumor did not respond to neoadjuvant chemotherapy in any patients. No significant difference was seen in relapse free survival or overall survival rates between the patients treated with adjuvant (two patients received a regiment cisplatin based) or neoadjuvant chemotherapy and those not treated. They conclude that SqCC of the breast is an extremely aggressive disease and better systemic treatment is required to prevent recurrence [8].

These findings are in accord with Rostock et al. review suggests that SqCC is not sensitive to chemotherapeutic agents commonly used for ductal carcinoma such methotrexate, cyclophosphamide, 5-fluorouracil (5-FU) and antracycline [12]. A good response on metastatic disease has been reported in one patient who received cisplatin and 5-fluorouracil but this has never been investigated in other report [15].

Our patient received adjuvant chemotherapy with 5-fluorouracil (5-FU) and cisplatin based on other experiences derived from some reports [1,3,4,15].

The role of radiation has been reported as unclear in many studies. Although squamous cell carcinomas are generally radiosensitive, locoregional relapse occurred frequently also in irradiated field. It seems that SqCC of the breast is often relatively radioresistant [3,4,8,9]. However, considering the conservative surgery performed, we proposed radiation therapy but the patient rejected.

Currently twenty-eight months passed from the surgery without evidence of disease.

## Conclusion

Pure Primary Squamous cell carcinoma of the breast is a very rarely and aggressive disease. Poor response of the SqCC of the breast to chemotherapeutic regimens commonly used in breast cancer, suggests that EGFR inhibitors and platin based regimens could be a promising option for treatment of these tumors. In our report the choice of adjuvant therapy was perfectly in accord with the new suggestions coming from recent literature. Clinical trials including large series of these rare tumors are needed to increase our knowledge and to improve patient's outcome.

## List of abbreviations

CT: Computed Tomography; ER: Estrogen receptor; SqCC: Squamous cell carcinoma.

## Consent

Written informed consent was obtained from the patient for publication of this case report and accompanying images. A copy of written consent is available for review by Editor-In-Chief of this journal.

## Competing interests

The authors declare that they have no competing interests.

## Authors' contributions

RM conceived the idea of the report and wrote the manuscript. DB reviewed the literature and wrote the manuscript. YM made the pathological diagnosis and performed immunohistochemical EGFR evaluation. LT reviewed the international literature and revised the manuscript. AB interpreted the findings, revised critically the manuscript and gave the final approval of the version to be published. FM performed wide local excision of the left breast with ipsilateral axillary lymphonodes dissection. All authors read and approved final version of manuscript.
